# Segmentation of Variants of Nuclei on Whole Slide Images by Using Radiomic Features

**DOI:** 10.3390/bioengineering11030252

**Published:** 2024-03-04

**Authors:** Taimoor Shakeel Sheikh, Migyung Cho

**Affiliations:** AIMI—Artificial Intelligence and Medical Imaging Laboratory, Department of Computer & Media Engineering, Tongmyong University, Busan 48520, Republic of Korea; sheikh@tu.ac.kr

**Keywords:** radiomics, deep learning, whole slide imaging, feature selection, classifiers

## Abstract

The histopathological segmentation of nuclear types is a challenging task because nuclei exhibit distinct morphologies, textures, and staining characteristics. Accurate segmentation is critical because it affects the diagnostic workflow for patient assessment. In this study, a framework was proposed for segmenting various types of nuclei from different organs of the body. The proposed framework improved the segmentation performance for each nuclear type using radiomics. First, we used distinct radiomic features to extract and analyze quantitative information about each type of nucleus and subsequently trained various classifiers based on the best input sub-features of each radiomic feature selected by a LASSO operator. Second, we inputted the outputs of the best classifier to various segmentation models to learn the variants of nuclei. Using the MoNuSAC2020 dataset, we achieved state-of-the-art segmentation performance for each category of nuclei type despite the complexity, overlapping, and obscure regions. The generalized adaptability of the proposed framework was verified by the consistent performance obtained in whole slide images of different organs of the body and radiomic features.

## 1. Introduction

Currently, the segmentation of multiple histological nuclei regions remains challenging for pathologists because of highly complex whole slide image (WSIs) datasets, which include intraclass variations and interclass similarity characteristics of nuclei cells. These parameters are critical factors and can provide clinically meaningful information during disease diagnosis [1]. However, the manual identification and inspection of various types of nuclei is a time-consuming task because of the morphological (size, shape, and structure), texture, and staining features of the nuclei regions and can result in variability among pathologists. Thus, the workflow for the diagnostic treatment of patients differs considerably. Therefore, an automated system that can perform quick WSIs scanning and segmenting of various nuclei variants should be designed to improve the workflow efficiency of pathologists.

The major challenges in learning or segmenting distinct types of nuclei from WSIs are the availability of accurately annotated data and the general limitations of WSIs, including imperfect slide preparation, staining complexity, overlapping nuclei, and artifacts of cellular structures in the histopathology data. Publicly available WSIs with accurate nuclear annotation information are rarely available, and acquiring such WSIs is difficult and expensive. To address this problem, we employed radiomics (quantitative) features that help to extract high-throughput information [2,3] from the patches of WSIs [4], and subsequent modeling using machine, and deep learning algorithms [5]. Existing learning methods exhibit considerable potential for solving general nuclei segmentation, but obtaining distinct and inferior quality multiple variants of nuclei from WSIs of different organs of the body using deep learning models is difficult because customized parameters are required for each experiment, preparing respective annotations is time-consuming, and/or configuration of training algorithms is difficult [6,7,8].

In this study, we proposed a framework to segment the various types of nuclei in WSIs using radiomic features that not only improve segmentation performance metrics but also help to learn low-intensity distinct nuclei regions. The proposed framework was based on variants of radiomic features (such as gray-level co-occurrence matrix features (GLCM), gray-level dependence matrix (GLDM), gray-level run-length matrix features (GLRLM), and gray-level size zone matrix (GLSZM)) that comprehensively learn the highly discriminative distributions and characteristics of each nuclei type and select the best sub-features of each radiomic using the LASSO operator. We then used multiple variants of machine learning classifiers (decision tree, K-nearest neighbors, bagging, and gradient boost) to retrieve the best patches. Subsequently, we trained different segmentation models using the retrieved patches to achieve the segmentation of various nuclei types. The aim is to use simple learning models that require a single unifying solution and less manual configuration. The contributions of this study are as follows:

1. The proposed framework improves the performance of different state-of-the-art (SOTA) segmentation models that learn and segment the type of nuclei regions in the WSIs that exhibit different characteristics.
2. The framework exhibits superior performance results for segmentation models, yielding the best score for each nuclei type using different metrics and achieving high metric scores for the public MoNuSAC2020 benchmark.
3. We demonstrated the performance of the framework from various perspectives using multiple radiomics types, loss functions, and visualization experiments. The results confirmed that our model was adaptable for diagnosing and segmenting different variants of nuclei.

The remainder of this paper is organized as follows: In Section 2, we discuss related research. The architecture and technical details of the proposed framework are presented in Section 3. Section 4 describes the dataset used in the study. The experimental setup, model training, and implementation details are described in Section 5. Section 6 provides a detailed comparison of the segmentation, visualization results of the segmentation patches, and ablation studies using the proposed framework. Finally, Section 7 provides the concluding remarks and potential areas for future research.

## 2. Related Work

Radiomics [9] features were used to extract tumor patterns and quantitative characteristics from WSIs by utilizing each feature information to segment the nuclei of different types that are otherwise not diagnosable (observable) through conventional algorithms for analysis [10,11]. An accurate inclusive segmentation process for various nuclei types is cumbersome because of the invariant and complex regions across different WSIs. By improving the quantitative analysis of WSIs using the automated high-throughput extraction method [12], the precision in diagnosis and assessment of prognosis increases, and decision-making is improved. However, several challenges have been reported [13,14].

Yuan [15] designed various radiomic models based on CT images to predict adenocarcinoma or squamous cell carcinoma in histopathology [16], PET tumor stage of lung cancer, and micropapillary patterns of lung adenocarcinomas using radiomics [17]. Liu [18] applied radiomics with different CNN models for Alzheimer’s disease diagnosis. In this method, several landmarks were used as extracted patches (normal and abnormal), and output decisions were made based on majority voting among all CNNs. Wang [19] reported the use of radiomics combined with machine learning to create a T-staging model for locally advanced laryngeal cancer; the AUC performance of the model was 0.892%. Choi developed an SVM-LASSO radiomic model to predict the malignancy of pulmonary nodules and improved classification using two radiomic features. The model achieved an accuracy of 84.6% [20]. Ning proposed a hybrid structure that includes various features selected with a radiomics model and CNNs and subsequently integrated these features to help classify patterns for gastrointestinal stromal tumors [21]. Urban designed a model based on radiomics to effectively detect and classify malignant polyps in WSIs [22]. Guo [23] designed a 3D deep learning (ProNet) model with radiomics (adNet) methods to automatically classify lung cancer into three types (distinguish lung adenocarcinoma, squamous cell carcinoma, and small cell lung cancer).

Using radiomics features have some limitations that affect the development of efficient diagnostics models, and treatment plans for individual patients [24,25]. Regardless of the potential of radiomics results, the repeatability, and reproducibility of radiomics features are still an important concern and often depend on the used sequence, size of the image, imaging modality and quality, as well as motion artifacts factors, which are commonly exploited during the extraction of features using acquisitions of images under identical or near-identical acquisition and processing parameters [26]. Some researchers made an effort to overcome the mentioned drawbacks which are ongoing in radiomics topic [27,28]. A recent review performed an extensive literature search and identified radiomics features that were shown to be repeatable and reproducible among the investigated studies [29].

Literate studies analyzing WSIs using radiomics have relied on predefined assumptions or learned features of nuclei or cancer types; however, because of high variants of samples or specific categories, these approaches are not feasible for segmentation owing to the non-availability of data on nuclei types. This problem limits the procedures and restricts the generalized adaptability of methods. Previous segmentation models [30,31,32] have achieved better performance; however, in the case of various nuclei types, a specific set of optimal hyperparameter tuning is required, or these models are more dependent on the segmented ROI of nuclei regions and regular nuclei types extracted from WSIs. By contrast, our research obtains segmented individual nuclei region information from WSIs by adopting variants of independent radiomics features of nuclei types using various classifiers, and different segmentation models that achieve improved performance for any type of nuclei. The selection of different radiomic features learned with the best classifier descriptors provides considerable discriminative information, regardless of complexity, and obscures the nuclear types of WSIs. The proposed framework comprises separable modules that can be used for various medical image analysis tasks.

## 3. Proposed Framework and Architecture

**Figure 1:**
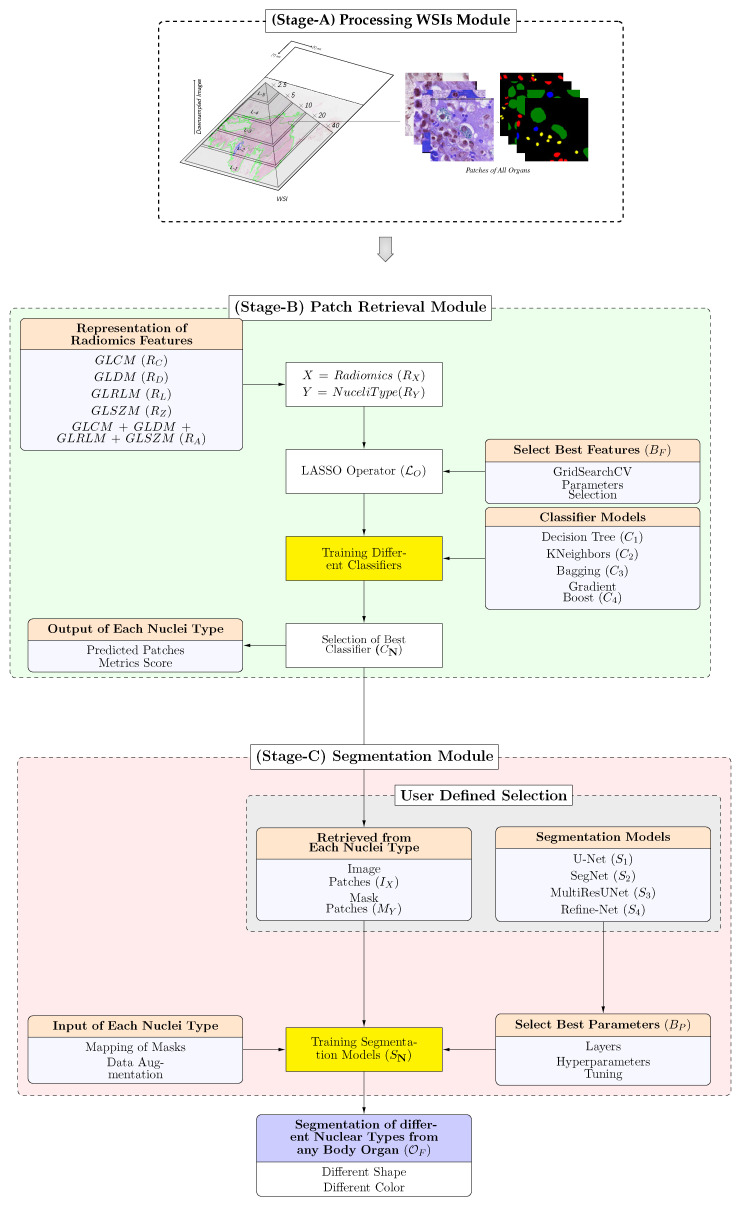
Block diagram of the proposed framework. (Stage-A) Segmentation based on annotations and generating image patches from the tissue regions of the WSIs. (Stage-B) Patches are encoded into a set of radiomic features (i.e., GLCM, GLCM, GLRLM, and GLSZM). The LASSO operation was applied to determine optimal sub-features from each radiomic feature. These features are passed separately, and in a fused form to the classifier with respective labels to train various machine learning classifiers, and based on the best classifier, the best patches and metric scores are retrieved. (Stage-C) For segmentation, the set of best retrieved images and masks are inputted to different segmentation models to learn individual variants of nuclei which are used to perform the final diagnostic segmentation.

In this section, we describe the pipeline of the proposed framework in Figure 1. The proposed framework included the following modules: (Stage-A) processing of the WSIs module that extracts patches from a dataset; (Stage-B) a patch retrieval module, in which different types of radiomic features were used to learn the inherent characteristics of different types of nuclei regions. Finally, the selected features were inputted into machine learning classifiers [33] to retrieve patches from the best classifier model; (Stage-C) the segmentation module includes different models [34,35], which are trained with the retrieved patches to segment the variants of nuclei regions.

### 3.1. (Stage-A) Processing WSIs Module

A novel WSI processing module was proposed. In this model, WSIs from the dataset were used with the corresponding XML file (mask), which includes relevant information on each type of nucleus (such as location and type). Initially, the WSIs of various organs of the body were read into memory at a downsampled dimension of WSI, for example, zero level, because all slides were from different sources, which affected the patching process. Therefore, to ensure the consistency of pixel locations for patches, we reduced and saved each slide in the tiff form of size 256 × 256 for easy visualization with its respective XML (masks) on the screen for segmenting and annotating nuclei-type regions.

The proposed module created patches by reading each tiff slide image and the binary mask values of the individual nuclei tissue regions, which are computed based on the nuclei type provided in the XML file by the organizers. The approximate contours of the detected foreground objects were then filtered based on an area threshold, and the segmentation mask for each slide was stored and saved in the same format as that of the original slide. To create patches, we adopted a sliding window operation with two crucial parameters, namely step size and patch size, which controlled the patching criteria. We used a patch size of 128 × 128 and a step size of 200 to store the stacks of image patches according to the nucleus class types using the normal jpg format. The sliding window operation was used because it creates overlapped regions as all nuclei sizes are so small that overlapping region information is required to obtain more samples for each type of nucleus. Depending on the size of the nuclei in WSIs, the number of patches extracted from each slide varied from hundreds to hundreds of thousands. The number of extracted patches for each nucleus class was saved in separate folders using the respective class-wise label information.

While training, our aim was not only to select the best classifier from different classifiers but also to analyze characteristics of the nucleus class with improved time and space complexity, so we used 28 × 128 patch size because it produces faster processing results with our GPU processing memory. We tried different sizes during our experiments but this patch size works perfectly. The image patch size and number of training samples together have a complex influence on the performance of the classification. Step size is variant but with some observations of created patches using various values, a step size of 200 value provides more samples for each type of nucleus, and with less overlapped regions for all nuclei sizes.

### 3.2. (Stage-B) Patch Retrieval Module

This module of the framework retrieves the best nuclei patches by training various machine learning classifiers that distinguish the four types of nuclei based on different radiomics (single or combination) features. We applied LASSO operation on each radiomics before training classifiers to increase the training efficiency time and selection of the best sub-features.

#### 3.2.1. Representation of Radiomics Features

After extracting patches from WSIs, we obtained a set of five radiomics features of these patches to learn the overall and discriminative variants between nuclear region types, such as shape, texture, and distances between regions. We used four radiomics techniques, namely $GLCM\;(R_{C})$, $GLDM\;(R_{D})$, $GLRLM\;(R_{L})$, $GLSZM\;(R_{Z})$. These four features help learn variants of information, especially for problems in which tissue heterogeneity in the nuclei regions of WSIs plays a crucial role because of the relationships between neighboring pixels [36]. This procedure was repeated for each organ of the body patch sample to generate nuclei-wise input data $X=Radiomics(R_{X})$ and respective nuclei class labels $Y=NuceliType(R_{Y})$. The radiomics features used in our framework are summarized in Table 1. Most of these features provide useful information based on matrixes derived from the intensity and correlation relationships between different pixels in a given 2D image.

1. Gray Level Co-occurrence Matrix (GLCM): This feature describes the spatial distribution of gray-level pixel intensities within a 2D image [37]. Features extracted from GLCM are calculated based on two predefined parameters Θ and d, where Θ ∈ 0°, 45°, 90°, and 180°, and d is any integer distance admissible within the image dimensions.
2. Gray Level Run Length Matrix (GLRLM): This feature calculates the number of pixels with the same gray and characterize the gray-level run lengths of different gray-level intensities in any direction [38].
3. Gray Level Size Zone Matrix (GLSZM): This feature quantifies the gray-level zones, that is the number of connected pixels that share the same gray-level intensity, in a 2D image [39].
4. Gray Level Dependence Matrix (GLDM): This feature includes the number of connected pixels within a distance dependent on the center pixel [40].

Because some extracted radiomic features can be irrelevant to nuclei types, many features in the semantic feature maps are highly redundant, which may negatively affect the classification or prediction results; therefore, we applied an additional operator, as explained in Section 3.2.2.

#### 3.2.2. LASSO Operator

Conventionally, 70 features are extracted from each type of radiomic feature $R_{X}$ to learn the respective discriminative features for each input patch. However, because of the distinct nature of each nuclei cell type from different sources of WSIs, we adapted the LASSO operation to select the best set of sub-features from each individual or combined radiomic feature. Table 1 presents a set of different sub-features in each radiomic feature; for example, if we select any radiomic feature $R_{X}$, it includes a different number of characteristics, represented as $R_{X}={\left\{Features_{i}\left(x\right)\right\}}_{i=1}^{n}$, where $Features_{i}\left(x\right)$ represents the sub-features corresponding to the respective radiomic features selected. To select relevant sub-features for a particular nuclei type from the entire radiomic feature, we applied the LASSO operator, which exhibits particular distributions or characteristics, rendering the model easier to handle by shrinking data values toward a central point, such as the mean of pixel values. Because of this characteristic, this property was the backbone of our patch retrieval module. Therefore, we defined the LASSO operator as follows:

(1) $$\begin{array}{c} L_{O}=\left\{Radiomics\;\left(R_{X}\right)\right\}=\left\{Features_{1}\left(x\right),Features_{2}\left(x\right),\ldots ,Features_{n}\left(x\right)\right\} \end{array}$$

The LASSO operator $\left(L_{O}\right)$ model was trained using five-fold cross-validations, and grid search $\left(B_{F}\right)$ to select the best feature sets. This phenomenon generated a refined subset of radiomics features $R_{X}$ for each nuclear type: $R_{C}$, $R_{D}$, $R_{L}$, $R_{Z}$, and $R_{A}$. Generally, pathologists study nuclei regions from various orientations, and many variation factors and acquisition conditions exist. We applied various radiomics combinations to learn realistic variations for pathological examination. These features, with their respective labels, were used to train the next part of the patch retrieval module, which was used to distinguish the four types of nuclei. The same procedure was repeated for each radiomic feature.

#### 3.2.3. Training Different Classifiers

To retrieve the best patches from the original patches, we used the best sub-features selected by the lasso operator of each radiomic to train four machines learning classifiers (such as decision tree: $C_{1}$, KNeighbors: $C_{2}$, Bagging: $C_{3}$, gradient boost $C_{4}$). So, for training each classifier, $C={\left\{R_{X_{i}}^{\theta_{i}}\right\}}_{i=1}^{n}$, parameterized by $\theta_{i}\in \Theta$, where Θ is the set of hyperparameters for each classifier model, and $R_{X_{i}}$ represents each radiomic feature. In the following section, we suppress the parameters from the notation of each classifier to simplify the explanation. The objective of each classifier is to predict input $R_{X_{i}}={\left\{Features_{i}\left(x\right)\right\}}_{i=1}^{n}$ and learn the effective nuclei characteristics based on the subset of each radiomics feature with the respective labels. During the training of each classifier, we minimized the loss function, which can supervise the relevance of the learned features and enhance their expressive ability.

#### 3.2.4. Selection of the Best Classifier

We used four types of metrics, and the results revealed that the features learned with these classifiers achieved the best classification performance, as discussed in Section 6.1. These training parameters were learned through back-propagation of the training phase and a cross-validation technique. In the final stage of this module, based on the performance metrics of the classifiers, we selected the best classifier, **$C_{N}$** which shows the highest metrics score, and the learning capability for each nuclei types, irrespective number of patch samples.

For each type of radiomic, the respective test subset of a refined subset of radiomics features $X=Radiomics\;(R_{X_{i}})$, and the labels $Y=NuceliType\;(R_{Y_{i}})$ were passed to the best classifier to classify the nuclei type-wise samples. We also saved the best (correct) sample indices and neglected the incorrect samples based on learned characteristics. We then compared these best indices with the original patches extracted from the WSIs to retrieve the best patches for use as inputs for the segmentation models. Details regarding our experimental setup are presented in Section 5. Our model has a loosely coupled architecture that allows switching between various classifiers without relearning the LASSO operator $\left(L_{O}\right)$ learned subfeature representation for each radiomic for training and retrieving the best image patches from the classifiers.

### 3.3. (Stage-C) Segmentation Module

#### 3.3.1. User-Defined Selection of Model

This module of the proposed framework segments the variants of nuclei-type patches and adapts the mapping of masks with data augmentation (if required) applied to the patches $X=Images\;(I_{X})$ and respective masks retrieved based on indices information $Y=Mask(M_{Y})$ retrieved from the previous module results. The segmentation model was selected by the user from the following two modules (such as U-Net: $S_{1}$, SegNet: $S_{2}$), and five cross-validation techniques using grid search $\left(B_{P}\right)$ were applied to select the best hyperparameters and layers for training each model separately for each radiomic case and for different kinds of nuclei.

#### 3.3.2. Training Different Segmentation Models

When we passed the set of patch results retrieved from the previous module of each nucleus as inputs to the segmentation model **$S_{N}$**, which helped the model learn and extract complex and obscure features in the nuclei patched images and prevented the model overfitting $\left(O_{F}\right)$. This module learns the importance of each nuclei type and suppresses the features that are less important to our nuclei segmentation task for particular nuclei types by minimizing the loss functions that improve segmentation performance. However, both these models in the literature exhibited the capability to learn well and significantly reduce the model complexity by improving the experimental performance, as presented in Section 6.2.

## 4. Dataset

**Figure 2:**
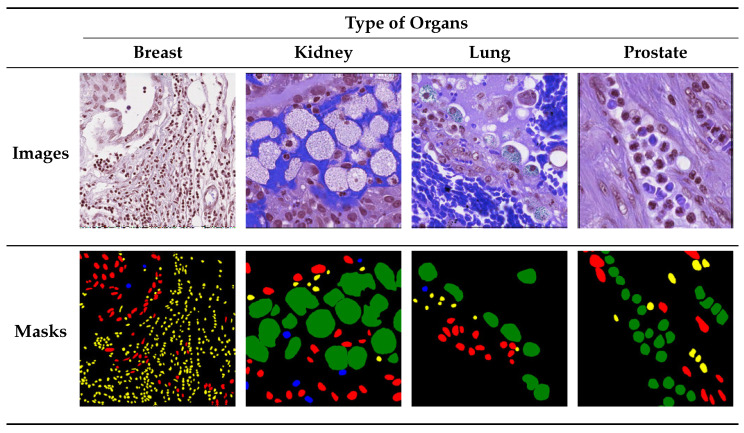
MoNuSAC2020 dataset samples: Sub-images, and annotations with boundaries of various types of nuclei shown with different colors: Epithelial cells in red, Lymphocyte in yellow, Macrophage in blue, Neutrophil in green, and Background in black.

The following public digital pathology dataset was used as the reference standard for training and validating the performance of the proposed framework. This dataset contains variants of nuclei from different organs. The MoNuSAC2020 [41] (publicly available at https://monusac-2020.grand-challenge.org; accessed on 10 April 2023) dataset includes two subsets, namely training and testing datasets. The training subset includes the WSIs of 46 patients from 32 hospitals downloaded from the (TCGA) data portal (The cancer genome atlas (tcga), http://cancergenome.nih.gov/; accessed on 10 April 2023). The WSIs were scanned by contributors at 40× magnification. Cropped WSIs ensured that the annotations were diverse and of high quality because we could sample more slides and nucleus-rich regions. Nucleus boundary annotations and class labels of each nucleus were adopted as previously established protocol and as described in [42]. The testing subset was prepared using the same procedure as the training subset described in [42]. Furthermore, the test data contained annotations of ambiguous regions. We used our processing WSIs module to generate patch-wise images from each WSI based on the nuclei-wise annotations of WSIs and categorized them into one of four classes, namely (1) epithelial, (2) lymphocytes, (3) neutrophils, and (4) macrophages, where the assigned class labels corresponded to a predominant nuclei type in the respective WSI, as displayed in Figure 2.

The objective of this dataset was to develop and compare robust algorithms for nuclear instance segmentation and classification into four nuclear classes. The distribution of nuclear types and organs is presented in Table 2. Since the number of nuclei information varies due to the structure of WSIs which are gathered from different sources, in every WSI the samples of epithelial, and lymphocyte types are approximately 10 times larger than the number of patches in the other two classes, and there was serious data imbalance. This imbalance in the dataset affected the performance largely for other classes. Therefore, we performed experiments on different WSIs and selected a random number of labeled WSIs, from which we generated a distinct number of patch samples for each organ of the body.

## 5. Experimental Setup

### 5.1. Performance Evaluation Criteria

We evaluated the performance of the proposed framework from two aspects: classifying nuclei samples based on radiomics and comparing them with literature segmentation models, as described in Section 6. The framework using five-fold cross-validation on a subset of samples was used to determine the best hyperparameters for each framework and module. We used multiple solid-state drives and hard drive storage to store the raw files of the digital WSIs. To perform patching, learning, and segmentation of WSIs, we used Intel Xeon CPUs, and feature extraction and learning were performed using an NVIDIA Quadro RTX 5000 on local workstations with 128 GB RAM. The proposed framework pipeline was implemented in Python 3.6, and uses image-processing libraries such as OpenSlide, OpenCV, and NumPy. We used the Keras deep learning library to load the data and train our framework. The source code is available at https://github.com/AIMILab/Radiomics, accessed on 26 February 2024).

### 5.2. Best Hyperparameters

The proposed framework comprises three modules. For the WSI processing module, three hyperparameters, namely patch size, step size, and area threshold value, were applied. The following hyperparameters were tuned for the classifier in the patch retrieval module: number of epochs, learning rate, batch size, and optimizer. The best-selected parameters of the classifier obtained using the cross-validation technique are listed in Table 3. For feature extraction from radiomics, we used the following four radiomics features with different hyperparameters that demonstrated the best results. These radiomics techniques help our patch retrieval module learn and classify input patch samples accurately with substantial information.

For the segmentation of various types of nuclei after retrieval of the best patches from the retrieval module, we used the 11 hyperparameters for tuning different segmentation models, and the best-selected parameters of each segmentation model obtained using the cross-validation technique are listed in Table 4. An additional parameter that was common to all settings was the mapping of masks on each nuclei type. The following different configurations of each model demonstrated the best results with and without radiomic feature samples. These segmentation models help to learn and segment the overall structures of different input patch samples of nuclear types accurately with substantial information.

### 5.3. Performance Evaluation Metrics

The performance of the proposed framework was evaluated using several metrics, including accuracy, sensitivity, precision, IoU score, and dice-coefficient. Equations (2)–(6) were used to compute these metrics, where the true positive (*TP*) is the number of correctly predicted samples as the positive class. False positive (*FP*) represents the number of incorrectly predicted samples as the positive class, and true negative (*TN*) represents the number of correctly predicted samples as the negative class. False negatives (*FN*) represent the number of incorrectly predicted samples as a negative class.

(2) $$Accuracy=\frac{TP+TN}{TN+FP+FN+TP}$$

(3) $$Sensitivity=\frac{TP}{TP+FN}$$

(4) $$Precision=\frac{TN}{TN+FP}$$

(5) $$IoUScore=\frac{TP}{TP+FP+FN}$$

(6) $$Dice-Coefficient=\frac{2\;\times \;TP}{2\;\times \;TP+FP+FN}$$

## 6. Performance Results and Discussions

### 6.1. Comparison of Different Classifiers

Table 5 presents the results of multiclass nuclei classification using the four SOTA classifier models for the MoNuSAC2020 dataset. The reported results revealed that radiomic features were added before each classifier learned the discriminative features, retrieved the best patches of each nuclei type, and demonstrated the highest metrics, regardless of the number of variants of samples and the shape of nuclei cells. The accuracy of the MoNuSAC2020 dataset increased to 82.1% when only the fused radiomic feature combination (All) was considered, and the gradient boost classifier performed significantly better than other classifiers for the MoNuSAC2020 dataset.

The gradient boost classifier for each radiomic feature also demonstrated the highest results for all other metrics when compared with other classifiers. The bagging classifier achieved the second-best classification results when the four classes were considered. Moreover, the fused radiomics feature combination (All) learns and classifies precise patches of nuclei that are more appropriate for diagnosing the types of nuclei, as demonstrated in our experimental results using each classifier. The metrics were not higher than 80% because in each WSI, the numbers of samples of macrophages and neutrophil classes were low, which affected feature learning from the classifier, and the distinction between classes was not sufficiently balanced. Another important challenge in radiomics is that a strategy for choosing the optimum architecture for a classifier is yet to be developed. Therefore, from the metric results, we can select the best classifier based on the highest metric score that is more efficient than other classifiers.

### 6.2. Comparison with Different Deep Learning Segmentation Models

**Figure 3:**
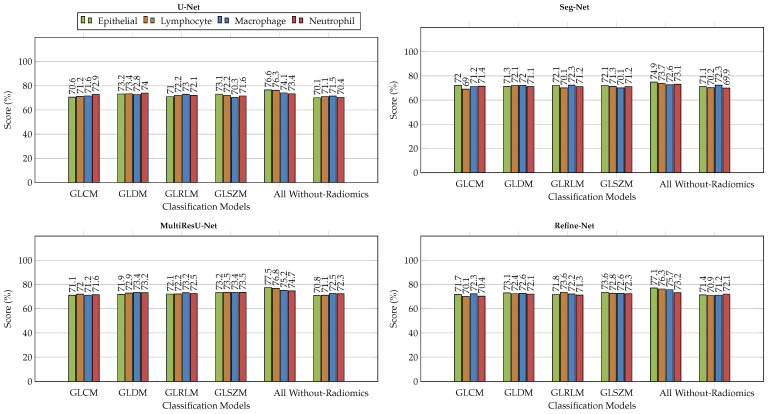
Comparison of accuracy score for multiclass nuclei segmentation of the MoNuSAC2020 dataset (best viewed in color).

**Figure 4:**
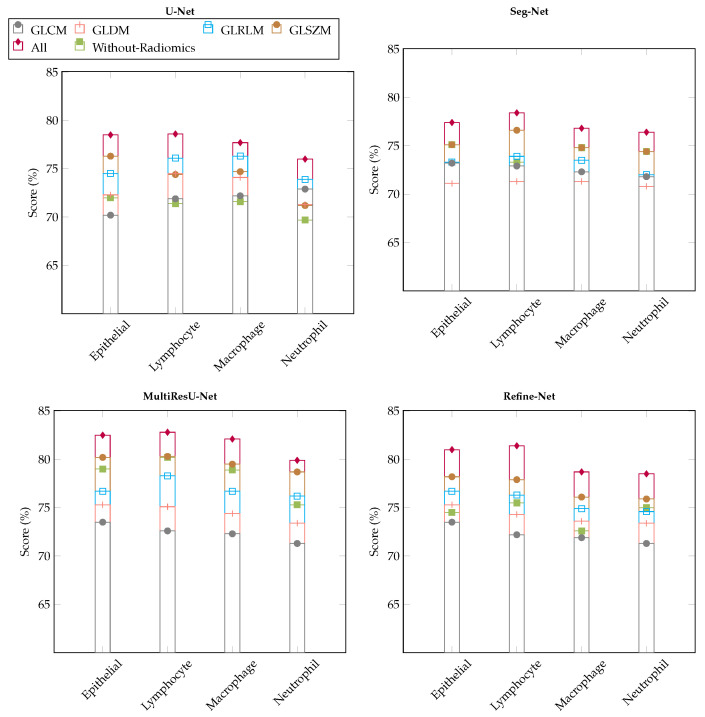
Comparison of sensitivity score for multiclass nuclei segmentation of the MoNuSAC2020 dataset (best viewed in color).

**Figure 5:**
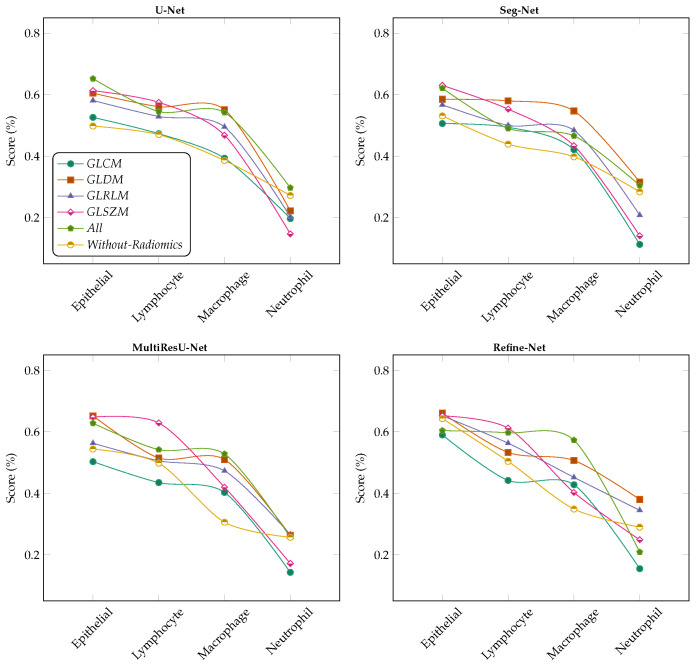
Comparison of IoU Score metric for multiclass nuclei segmentation of the MoNuSAC2020 dataset with different radiomic feature samples and without radiomic feature samples (best viewed in color).

**Figure 6:**
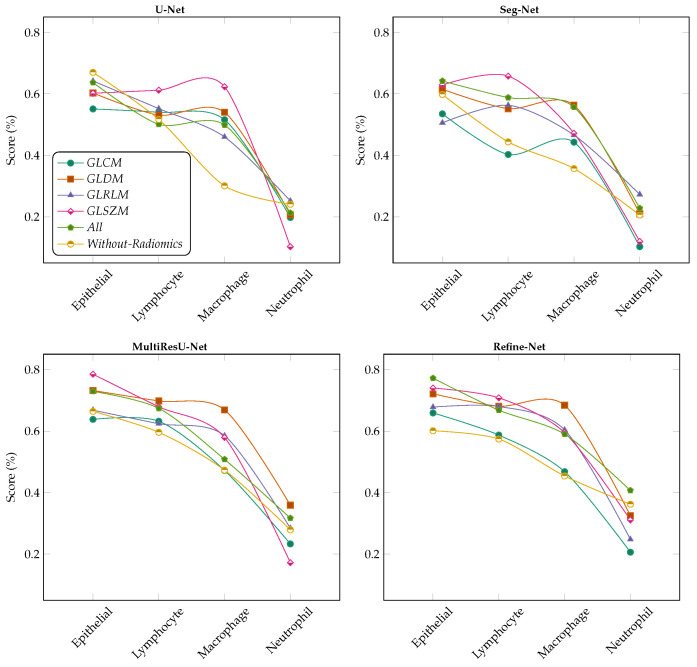
Comparison of Dice-Coefficient metrics for multiclass nuclei segmentation of the MoNuSAC2020 dataset with different radiomic feature samples and without radiomic feature samples (best viewed in color).

We demonstrated the experimental results for the MoNuSAC2020 WSI dataset using the segmentation of nuclei samples, with and without radiomic features as shown in the Figure 3, Figure 4, Figure 5 and Figure 6. We used four different segmentation models, namely U-Net [34], Seg-Net [35], MultiResU-Net [43], and Refine-Net [44]. Figure 3 demonstrates the accuracy metric results of the proposed framework for different, combined, and without radiomic feature samples with four types of segmentation models, which demonstrates an improved performance score by incorporating the patch retrieval module before the segmentation module, regardless of the variants of patch sample.

The accuracy increased to almost 77.0%, 75.0%, 78.0%, and 77.0% for all radiomic feature cases for the U-Net, Seg-Net, MultiResU-Net, and Refine-Net models, respectively, and 98.1% for the MoNuSAC2020 dataset. Similarly, the second highest accuracy is for the GLDM case for all segmentation models. Moreover, our proposed framework helps to retrieve and classify the robust patches because of the classifier techniques used with the LASSO operator. Therefore, the accuracy metric demonstrates that our framework can achieve much higher results from using any type of segmentation model regardless of the model structure. The reported results are improved because of the patch retrieval module added before each segmentation module to retrieve the best discriminative patches of each variant of nuclei subtype, regardless of the number of magnification levels of samples and the variants of nuclei subtypes. The accuracy of the dataset increased from 73.0% to 78.0% when all combined radiomic feature samples were considered, and the GLDM and GLSZM features performed notably better than other radiomic features.

Similarly, Figure 4 demonstrated the sensitivity metric results for the MoNuSAC2020 dataset using four different segmentation models. Our experiments show that higher scores are achieved for all radiomic feature samples as compared to the without radiomic samples, which exhibit the capability of our framework which introduced the patch retrieval module to correctly retrieve the best patches from the classifiers of each nucleus subtype with their respective classes. It should be observed that to make the patch retrieval module more functional we employed a lasso operator to improve the learning process than later simple applying classifiers for WSIs classification. From our results, we observed that the MultiResU-Net segmentation model assists efficiently in segmenting the best nuclei for the MoNuSAC2020 dataset and further improves the performance of performance each class type.

For the Refine-Net model, the segmentation results vary slightly too much due to the kernel regularizer parameter. In contrast, the results of the U-Net, and Seg-Net models are more accurately firm and consistent with and without the radiomic samples. The sensitivity metric results show that the nuclei subtype variants are classified well based on the retrieval module. The selection of patches depends on classifier training so few classes consist of more nuclei subtype regions respective to other classes in different magnification levels so, such variations affect the classifier selection criteria results. Regardless, the overall sensitivity metric results show that results can be improved with more hyper-parameter tuning of classifiers for selecting more approximate good sample patches with an even number of nuclei samples in WSIs to further enhance segmentation performance, indicating that the relevant patch retrieval for WSIs data is very effective.

In Figure 5 and Figure 6 the Refine-Net model demonstrated the highest IoU score, and dice-coefficient results for most radiomics cases, and even without radiomics cases. The Refine-Net model experiments consistently obtained higher scores than the other segmentation models, which demonstrated that the MultiResU-Net model learning capability is better for nuclei from complex regions and microstructure attributes, and correctly segments the nuclei images with their respective classes. We can state that using radiomic features with classifiers enhances the generalization capability and its independence from expert supervision, which is suitable for clinical pathology and for addressing the segmentation of each nuclei-type task within WSIs. The performance of each radiomics feature was compared with that of the segmentation model.

We replaced the conventional segmentation mechanism with training models using the best patches retrieved from the best classifier, which were trained on different radiomic features in the patch retrieval module. The accuracy of all experiments was reported using five-fold cross-validation. The maximum accuracies were obtained after fine-tuning the hyperparameters of the segmentation models. Thus, the numerical results of macrophages and neutrophils were low because the complex pathological data samples were neither sufficient in each WSI nor improved the performance results when using individual radiomics. The use of radiomics with segmentation obtained higher metrics than those without radiomics features learned for the MoNuSAC2020 dataset. The proposed patch retrieval module with the radiomic learning of the nuclei region is more effective than simple learning from direct nuclei regions in images.

The behavior of all models for multiclass nuclei segmentation was thoroughly studied, with a special emphasis on factors affecting performance. The types of radiomic inputs to the segmentation models significantly affected performance. Figure 5 and Figure 6 reveal the performance improved on passing different types of inputs to the classifiers in the form of radiomic features for the MoNuSAC2020 dataset. The input fused radiomic feature combination (All) shows the highest metrics, which indicates that each radiomic helps to learn different nuclei characteristics for segmentation. This phenomenon can affect the learning procedure by the given input radiomics. A single input of radiomics considerably degrades the results, as indicated by the input radiomic features. Performance can be improved using more radiomics as inputs, which boosts the metrics and classifies the samples more efficiently. Based on our experiments, we concluded that our results align with the design of using different radiomics feature combinations as inputs to the segmentation models, and the retrieval of sample patches from classifiers based on different radiomics features for segmenting the different variants of nuclei from the WSIs is more effective. We believe that these results can be applied in general medical imaging. From our observation, the results show that Refine-Net works best as compared to other segmentation models for our proposed framework.

### 6.3. Visualization of Segmented Samples

**Figure 7:**
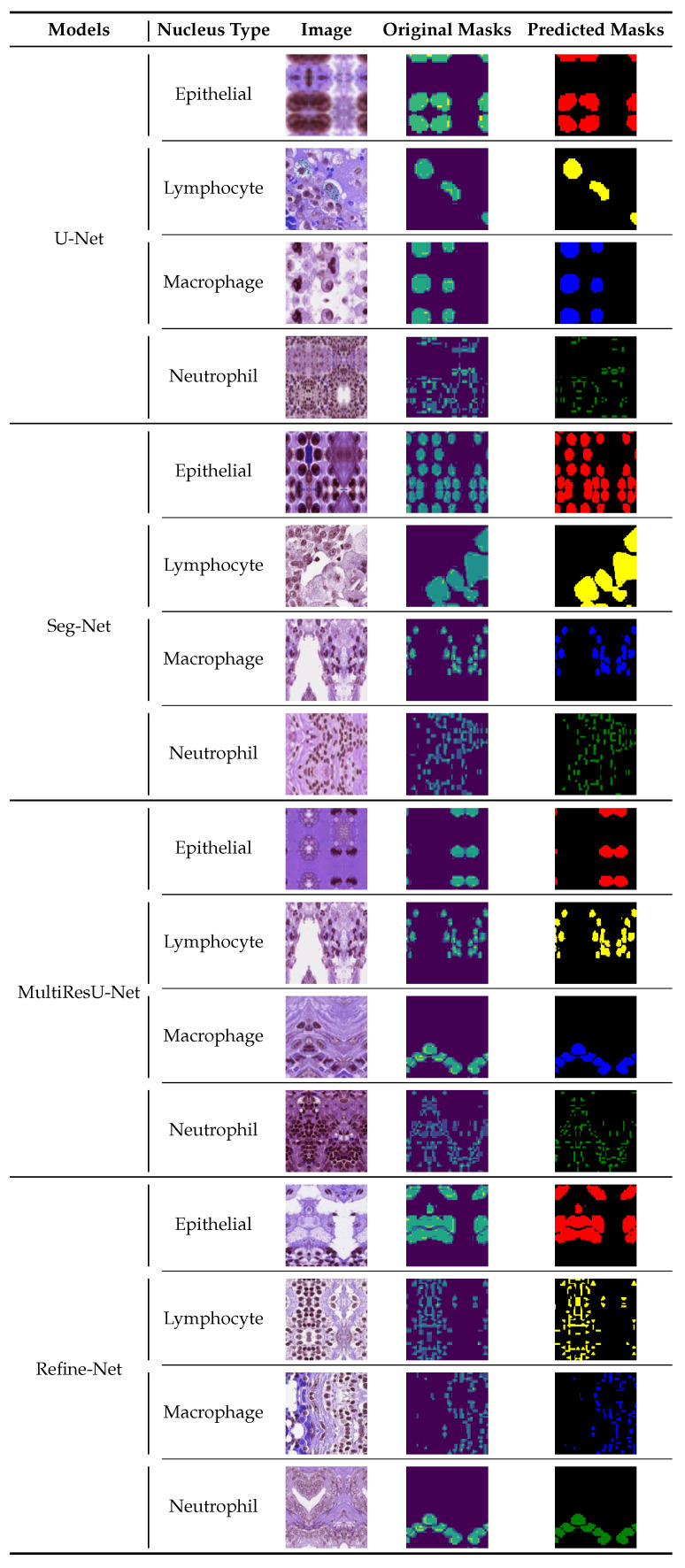
Best segmented patches of the MoNuSAC2020 dataset according to four kinds of nuclei when we pass the fused radiomic feature combination (All) for the training of different well-known segmentation models. Given the image on the left, we segmented the nuclei type to visualize a nuclei-affected or unaffected region (best viewed in color).

Figure 7 displays a visual illustration of the segmented patches of various types of nuclei learned from the two segmentation models when we used a fused radiomic feature combination (All) for the training models. These representations result from the different WSI patches, which indicate the effectiveness of using radiomics techniques for learning the unique characteristics of nuclei that help to learn individual kinds of nuclei. The visualization results revealed that each model makes a very accurate and meaningful segmentation of various variants of nuclei, irrespective of their morphology (size, shape, and structure), texture, and staining features of the nuclei regions. In each row, the first column represents patch images, the second column represents the original masks, and the final column shows the predicted masks with segmented nuclei regions for each class type, indicating excellent differentiation between each nuclei type with different colors.

We analyzed and observed that the results of the MultiResU-Net model were more appropriate than other segmentation models, the learning capability of microstructure attributes of the nuclei from the complex regions and different organs of the body, and correctly segmented the nuclei images with their respective classes. The Refine-Net model enhanced performance capability, which is more suitable for clinical pathology and addressing multitasking problems associated with WSIs. Therefore, the visualization results revealed that the U-Net model was more efficient than the other conventional segmentation models.

### 6.4. Ablation Studies

**Figure 8:**
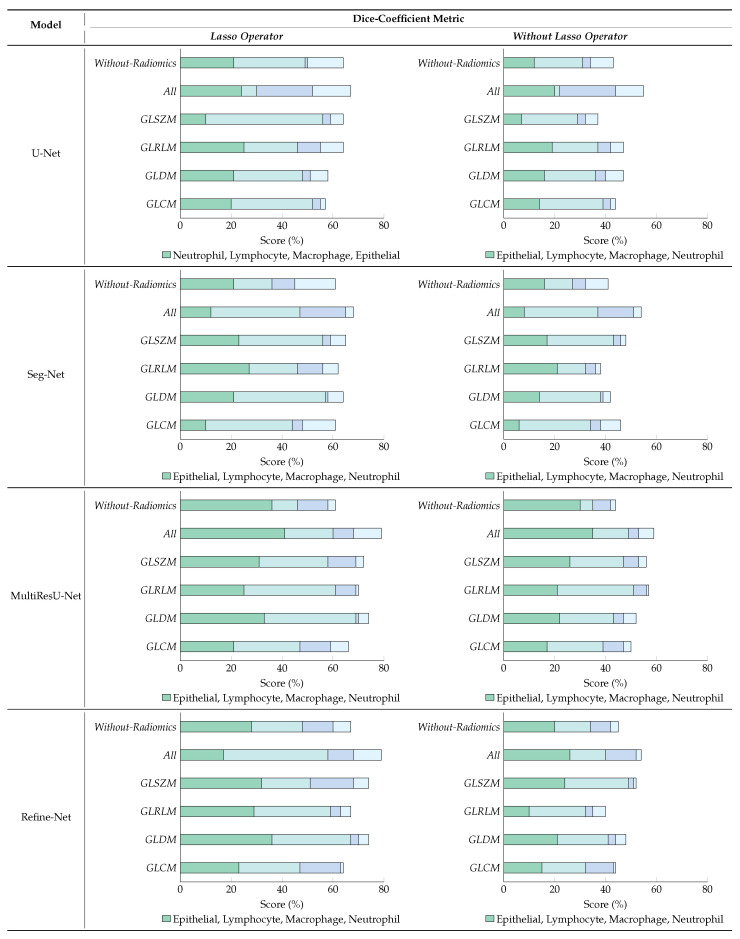
Results of ablation studies on the MoNuSAC2020 dataset using four multi-segmentation models, and by applying variants of radiomic feature samples with & without lasso operator (best viewed in color).

**Figure 9:**
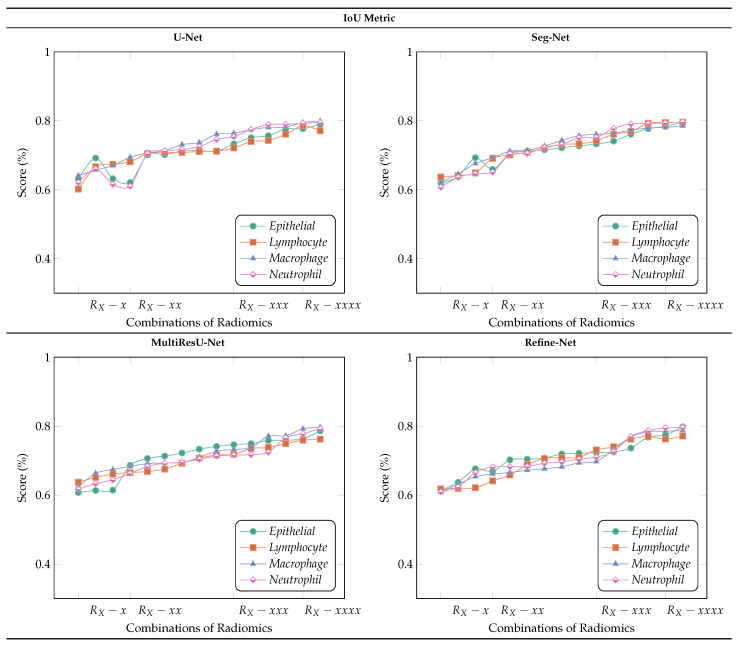
Results of ablation studies on the MoNuSAC2020 dataset using different multi-segmentation models by applying variants of radiomic feature samples. We select a different combination of radiomics to retrieve the best nuclei patches, i.e., $R_{X}-x$, representing pairs, triplets, quadruplets, and individual concatenated of different radiomic features. For example: $R_{X}-x$ or $R_{X}-xxx$, represents that we used only $R_{C}$ (GLCM) or $R_{C},R_{D},R_{L}$ (GLCM, GLDM, and GLRLM), respectively ((best viewed in color).

We demonstrated ablation studies using the proposed framework to yield deeper insights into the performance improvements associated with the different components within the framework. The problem of multi-class nuclei segmentation classification has been precisely observed, with a special emphasis on why we used radiomic features and effects on different segmentation models in our proposed framework. Figure 8 and Figure 9 show the results of the ablation studies, using Dice-Coefficient, and IoU metrics and different radiomic features-based patch samples.

#### 6.4.1. Effect of Lasso Operator

Figure 8 study shows that dice-coefficient metric variation with and without applying the lasso operator for the segmentation models, where we used different radiomic features (i.e., GLCM, GLDM, GLRLM, GLSZM, All, Without-Radiomics) patch samples retrieved from various machine learning classifiers and later used in segmentation models to distinguish the four types of nuclei patch samples of WSIs. For experiments, we compared the performance of our framework using four state-of-the-art segmentation models such as U-Net [34], Seg-Net [35], MultiResU-Net [43], and Refine-Net [44], because it yields the best performance in the literature. The results of our performance showed that training the models with different radiomic samples has significantly improved the overall performance compared to those without radiomic sample patches. The MultiResU-Net and Refine-Net models obtained the highest metric results while maintaining a stable segmentation using a fused radiomic feature combination (All).

Moreover, our results demonstrate that our method learns radiomic-based patch samples that are robust when we apply an additional lasso operator to select the best sub-set features of each radiomic input, as demonstrated by our results of different radiomic combinations. In contrast, all model experiment without a lasso operator shows that the metric results are reduced, which are much lower even for any kind or combination of radiomic samples as compared when we applied a lasso operator. We also observed a trend can be seen in that our method without a lasso operator does not perform well for Seg-Net, and U-Net by selecting radiomic sample patches, which yields lower metric results.

We believe that, in general, fused radiomic feature combination (All) patch samples can improve the model performance because it allows the trained model to have variant information of different kinds from different combinations of radiomic inputs. By increasing radiomic types, we can further increase the performance, so each model can accurately segment the nuclei sample regions. Please note that more combinations of radiomic inputs introduce variations for learning nuclei information, by introducing the number of diversity characteristics of data. Hence, by incorporating the patch and segmentation modules, each model demonstrates enhanced functionality to distinguish between the nuclei samples of different types, which are in general close enough to the realistic samples. The experimental results of the effect of the lasso operator show the validity of our framework utilizing different modules, which improves the metrics when we use the traditional segmentation method with the basic flat input without radiomic samples.

#### 6.4.2. Variants Effect of Radiomic Features Combinations

Figure 9 demonstrates the results obtained using variant combinations of radiomic feature samples to retrieve the best nuclei patches, and later used in segmentation models to distinguish the four types of nuclei patch samples of WSIs with different multi-segmentation models. The radiomics used are, i.e., $R_{C}$ (GLCM), $R_{D}$ (GLDM), $R_{L}$ (GLRLM), and $R_{Z}$ (GLSZM). For example, $R_{X}-x$ or $R_{X}-xxx$, represents that we used only $R_{Z}$ (GLSZM) or $R_{D},R_{L},R_{Z}$ (GLDM, GLRLM, and GLSZM), respectively.

We observed that almost all models achieved better metric performance when we used two or more different radiomic feature samples in combined form, which helped in segmenting nuclei samples more accurately using rich information learned by patches, compared with the scenario in which only individual radiomic samples is employed, as shown in Figure 9. The MultiResU-Net model shows better performance than compared with other multi-class segmentation models, with an overall average score of over 80%. We also observed that there is a trend in IoU metric scores there are several changes for each type of nuclei and type of segmentation model when fewer combinations of radiomic samples are employed. This is due to the following factors: radiomics with fewer than two combinations are not very useful for nuclei segmentation in WSIs because of large combinations of intra-class variations in nuclei information, which also affect the capability of classification models in retrieving patches with their respective classes.

Nevertheless, our framework still achieves a comparable performance with different segmentation models when we use combinations of radiomic feature-based patches with more than pair combinations. Hence, we can say that the segmentation models when used with more combinations of radiomic feature patches are more suitable and accurate for nuclei segmentation and addressing WSIs multi-class nuclei problems. However, from the experiments, we can state that the selection of radiomic feature-based patches affects the results in various ways, but as already explained, in general, there is an improvement with higher combinations.

## 7. Conclusions

In this study, the proposed framework improved segmentation performance for the nuclei types of distinct categories from different organs of the body by using a radiomics technique. Four variants of radiomics features that learn quantitative information about each type of nuclei were used. Next, based on radiomic information, we trained different classifiers. Subsequently, we passed the outputs of the best classifier to different segmentation models to learn and segment the nuclei. We used the MoNuSAC2020 dataset and achieved SOTA segmentation performance using two models for each type of nucleus extracted from the WSIs. We also analyzed the efficiency of the results when we did not apply radiomics features. The experimental results demonstrated that the framework achieved promising performance and can play a critical role in the diagnosis of any type of nuclei region from WSIs. The proposed framework can use any number of independent radiomics, which enhances the model’s generalized adaptability to learn variants of nuclei from different WSIs.

The limitation of the proposed framework is the size of the patches, which can affect the training efficiency of classifiers; a larger patch size requires larger memory for conversion into radiomic features and subsequent training in the retrieval and learning stages. In the future, we intend to develop a robust patch retrieval module by adding deep learning models (such as self-supervision or transfer learning) for feature extraction from WSI patches. We will investigate the performance of the proposed framework on other radiomic features that provide variant comparisons and other datasets that provide diverse nuclei cases. The proposed system can be adapted to diverse tasks associated with the domain of WSI-based diagnosis with relevance to clinical settings.

## Figures and Tables

**Table 1:** Radiomics features used to classify the types of nuclei in the patch retrieval module.

GLCM (24)	GLDM (14)	GLRLM (16)	GLSZM (16)
Autocorrelation	Dependence Entropy	Gray Level Non-Uniformity	Gray Level Non-Uniformity
Cluster Prominence	Dependence Non-Uniformity	Gray Level Non-Uniformity Normalized	Gray Level Non-Uniformity Normalized
Cluster Shade, Joint Entropy	Dependence Non-Uniformity Normalized	Gray Level Variance	Gray Level Variance
Cluster Tendency, Joint Average	Dependence Variance	High Gray Level Run Emphasis	High Gray Level Zone Emphasis
Contrast, Joint Energy	Gray Level Non-Uniformity	Long Run High Gray Level Emphasis	Large Area Emphasis
Correlation, Inverse Variance	Gray Level Variance	Long Run Low Gray Level Emphasis	Large Area High Gray Level Emphasis
Difference Average, Sum Average	High Gray Level Emphasis	Low Gray Level Run Emphasis	Large Area Low Gray Level Emphasis
Difference Entropy, Sum Entropy	Large Dependence Emphasis	Run Entropy	Low Gray Level Zone Emphasis
Difference Variance, Maximum Probability	Large Dependence High Gray Level Emphasis	Run Length Non-Uniformity	Size Zone Non-Uniformity
Inverse Difference	Large Dependence Low Gray Level Emphasis	Run Length Non-Uniformity Normalized	Size Zone Non-Uniformity Normalized
Inverse Difference Moment, Sum Squares	Low Gray Level Emphasis	Run Percentage	Small Area Emphasis
Inverse Difference Moment Normalized	Small Dependence Emphasis	Run Variance	Small Area High Gray Level Emphasis
Inverse Difference Normalized	Small Dependence High Gray Level Emphasis	Short Run Emphasis	Small Area Low Gray Level Emphasis
Informational Measure of Correlation 1	Small Dependence Low Gray Level Emphasis	Short Run High Gray Level Emphasis	Zone Entropy
Informational Measure of Correlation 2	-	Short Run Low Gray Level Emphasis	Zone Percentage
Maximal Correlation Coefficient	-	Long Run Emphasis	Zone Variance

**Table 2:** Composition of the MoNuSAC2020 dataset by organ and nuclei type. N: The number of WSIs is presented in **bold**, and the number of patches is shown in *italics*. A random number of selected WSIs, and generated patch samples for our experiments are shown in brackets. We used a patch size of 128 × 128.

Nuclei Type	Training Subset			
	Breast	Kidney	Lung	Prostate
	***N*** ** = 10 (7)**	***N*** ** = 12 (11)**	***N*** ** = 10 (12)**	***N*** ** = 14 (8)**
	*670 (2055)*	*566 (2477)*	*506 (2647)*	*570 (1021)*
Epithelial	4566 (540)	3547 (1501)	2981 (474)	3445 (575)
Lymphocytes	4689 (946)	4126 (259)	3018 (916)	3821 (275)
Macrophoges	147 (37)	102 (354)	186 (887)	152 (70)
Neutrophils	105 (43)	149 (250)	142 (281)	235 (55)
	**Testing Subset**			
	***N*** ** = 5 (4)**	***N*** ** = 7 (3)**	***N*** ** = 7 (3)**	***N*** ** = 6 (3)**
	*220 (725)*	*310 (860)*	*220 (670)*	*260 (610)*
Epithelial	1377 (415)	2248 (510)	1489 (478)	2099 (274)
Lymphocytes	1833 (258)	2141 (256)	1674 (128)	2158 (191)
Macrophoges	49 (32)	84 (56)	53 (42)	121 (106)
Neutrophils	26 (20)	56 (38)	36 (22)	54 (39)

**Table 3:** Best hyperparameters of best classifier for various types of nuclei.

Classifier	Parameters	Radiomics				
		GLCM	GLDM	GLRLM	GLSZM	All
Bagging Classifier	N Estimators	50	50	25	25	50
	Max Samples	1.0	1.0	1.0	1.0	1.0
	Max Features	1.0	0.5	0.5	0.5	1.0

**Table 4:** Best hyper-parameters of the segmentation models for the MoNuSAC2020 dataset.

Models	Parameters	Radiomics GLCM GLDM GLRLM GLSZM	All	Without-Radiomics
U-Net	Number of layers	41		
	Epochs	20	15	15
	Optimizer	Nadam		
	Batch Size	16	16	32
	Kernel Size	3, 3		
	Filters	16, 32, 64, 128, 256		
	# of Parameters	1.9 M		
	Kernel Regularizer	He Normal		
	Activation Map	Sigmoid, Relu		
	Dropout Rate	0.5		
	Learning Rate	10^−4^	10^−3^	10^−3^
Seg-Net	Number of layers	62		
	Epochs	15	20	15
	Optimizer	Nadam		
	Batch Size	16	16	32
	Kernel Size	3, 3		
	Filters	64, 128, 256, 512		
	# of Parameters	11 M		
	Kernel Regularizer	He Normal		
	Activation Map	Sigmoid, Relu		
	Dropout Rate	0.5		
	Learning Rate	10^−4^	10^−3^	10^−4^
MultiResU-Net	Number of layers	239		
	Epochs	15	20	20
	Optimizer	Adamax		
	Batch Size	16	32	32
	Kernel Size	3, 3		
	Filters	32, 64, 128, 256, 512		
	# of Parameters	7.2 M		
	Kernel Regularizer	l2		
	Activation Map	Sigmoid, Elu		
	Dropout Rate	0.5		
	Learning Rate	10^−3^	10^−3^	10^−3^
Refine-Net	Number of layers	480		
	Epochs	20	15	15
	Optimizer	Adamax		
	Batch Size	32	16	16
	Kernel Size	3, 3		
	Filters	64, 128, 256, 512, 256, 1024, 2048		
	# of Parameters	42 M		
	Kernel Regularizer	l1		
	Activation Map	Sigmoid, Elu		
	Dropout Rate	0.6		
	Learning Rate	10^−3^	10^−4^	10^−4^

**Table 5:** Composition of the MoNuSAC2020 dataset.

Radiomics Feature	Classifier	Metrics		
		Accuracy	Sensitivity	Precision
GLCM	Decision Tree	0.756	0.757	0.759
	KNeighbors	0.760	0.760	0.769
	Bagging	0.776	0.778	0.784
	GradientBoost	0.779	0.779	0.788
GLDM	Decision Tree	0.772	0.774	0.779
	KNeighbors	0.794	0.793	0.799
	Bagging	0.808	0.804	0.814
	GradientBoost	0.810	0.809	0.816
GLRLM	Decision Tree	0.780	0.789	0.781
	KNeighbors	0.803	0.801	0.808
	Bagging	0.805	0.809	0.807
	GradientBoost	0.819	0.810	0.825
GLSZM	Decision Tree	0.763	0.764	0.767
	KNeighbors	0.782	0.781	0.785
	Bagging	0.795	0.796	0.802
	GradientBoost	0.796	0.797	0.803
All	Decision Tree	0.780	0.781	0.783
	KNeighbors	0.803	0.802	0.807
	Bagging	0.816	0.817	0.821
	GradientBoost	0.821	0.820	0.826
Without-Radiomics	Decision Tree	0.774	0.787	0.749
	KNeighbors	0.796	0.759	0.765
	Bagging	0.785	0.736	0.742
	GradientBoost	0.784	0.691	0.764

## Data Availability

Publicly available datasets were analyzed in this study. This data can be found in [https://monusac-2020.grand-challenge.org] and [http://cancergenome.nih.gov] (accessed on 10 April 2023).
